# Accommodations for the Next Meeting of the American Institute

**Published:** 1894-09

**Authors:** 


### ACCOMMODATIONS FOR THE NEXT MEETING OF THE AMERICAN INSTITUTE.

The First Baptist Meeting-house, in Newport, R. I., has been engaged by the Local Committee of Arrangements, for the use of the American Institute of Homœopathy in June next. This is a plain, white, wooden structure of the type so frequently found in the rural districts of New England, having been erected nearly half a century ago. (The church itself was constituted in 1638.) Its sittings, however, are comfortable and probably ample, for one thousand persons can be accommodated in its audience-room without difficulty. This will be reserved for the use of the Section in Ophthalmology, Otology, and Laryngology for two full meetings. The large vestry is supplied with comfortable chairs, seating three hundred and fifty people at least, an attendance which few, if any, sections ever exceed. The small vestry, which has a separate entrance from the church-yard, as well as from the large vestry, can conveniently care for one hundred and fifty visitors (this is for sections holding sessions on the sly), while a committee room, large enough to receive the Senate of Seniors or the Inter-Collegiate Committee, will afford ample accommodations for the Treasurers and Registrar. The two most honorable bodies above referred to will probably be assigned special parlors at the Ocean House. Minor committees will be cared for there also. It will be noted that while there is sufficient space in this meeting-house to fulfill the demands of the Institute in its entirety as well as in its integral parts, there is no room within its walls for anything tending in the least to distract the members from the object for which they are assembled—the transaction of business pertaining to the Institute, and to the promotion of medical science. Social features will be provided for at the Ocean House.

